# Violence reduces attention to faces and draws attention to points of contact

**DOI:** 10.1038/s41598-019-54327-3

**Published:** 2019-11-28

**Authors:** Coltan Scrivner, Kyoung Whan Choe, Joseph Henry, Muxuan Lyu, Dario Maestripieri, Marc G. Berman

**Affiliations:** 1 Department of Comparative Human Development, The University of Chicago, Chicago, IL USA; 2 Institute for Mind and Biology, The University of Chicago, Chicago, IL USA; 3 Department of Psychology, The University of Chicago, Chicago, IL USA; 4 Mansueto Institute for Urban Innovation, The University of Chicago, Chicago, IL USA; 5 Grossman Institute for Neuroscience, Quantitative Biology, and Human Behavior, Chicago, IL USA

**Keywords:** Social behaviour, Human behaviour

## Abstract

Although violence is a frequently researched topic, little is known about how different social features influence information gathering from violent interactions. Regions of an interaction that provide contextual information should receive more attention. We predicted the most informative features of a violent social interaction would be faces, points of contact, and objects being held. To test this, we tracked the eyes of 90 participants as they viewed images of social interactions that varied with respect to violence. When viewing violent interactions, participants attended significantly less to faces and significantly more to points of contact. Moreover, first-fixation analysis suggests that some of these biases are present from the beginning of scene-viewing. These findings are the first to demonstrate the visual relevance of faces and contact points in gathering information from violent social interactions. These results also question the attentional dominance of faces in active social scenes, highlighting the importance of using a variety of stimuli and contexts in social cognition research.

## Introduction

As social primates, humans spend a large portion of their time around other humans, looking at other humans, and thinking about other humans. People use visual gaze to gather information about others, including their actions, intentions, and personality^1,2^. Enhanced social cognition, such as the ability to understand others as intentional agents, is one of the defining features of *Homo sapiens*^3^. It is in viewing social interactions that this ability is most explicitly expressed. Therefore, understanding how people gather and integrate visual information from social interactions is crucial for understanding human behavior in the real world.

Many visual stimuli compete for our attention, and gaze fixation must be directed to a region of a scene in order to acquire high-quality visual input^4^. However, our eyes can only fixate on one thing at a time and not all stimuli are equally important. To solve this ecological problem, we possess evolved tendencies to selectively fixate on certain stimuli to a greater extent than others. Human infants have an attention bias towards faces and objects being held by their caregiver, both of which convey important social information during early development^5,6^. Individuals of all ages exhibit attentional biases toward potentially dangerous visual stimuli, such as snakes^7^ and angry faces^8,9^. It has been postulated that these attentional biases exist because they increase efficient detection of evolutionarily important sources of threat^10,11^. Moreover, some of these attention biases may be evolutionarily conserved, as they have also been observed in other primates^12,13^.

With all of this knowledge, we still know remarkably little about how people visually assess social interactions. Some research has suggested that people allocate an overwhelming majority of their attention to eyes in social scenes^14^. Although gaze cues are an important source of information, it is unclear why people would require so much time to determine gaze cues since gaze direction can be quickly determined^15^. One possible explanation has to do with the type of stimuli used in previous studies. For example, previous studies on attention to social features in scenes either used images that contained a single person^16^, several people who were not interacting^17^, or more than one person interacting, but in a very minimal way^14^. Gaze patterns in these types of social scenes may not translate well to other social situations, especially where people are more actively interacting with each other, such as in a violent situation.

People display attentional biases towards violent and threatening information across several different domains, including rumors, media, news, and images^18–22^. Moreover, low level physical saliency appears to be less predictive of attention in social scenes and negative scenes^17,23,24^. This suggests that social scenes, and particularly negative or threatening social scenes, may elicit specific visual information gathering patterns relevant to their semantic content. Certain areas of threatening social scenes may provide information that is pertinent for understanding the context of an interaction with important social consequences. For example, witnessing interpersonal violence can provide an opportunity to gather socially critical information about aggressors and victims, potential coalitional partners, and changes in status, power, and hierarchy^25^. Consequently, research on the social cognition of violence could be relevant and important for both theoretical and applied research.

The current study is an analysis of visual fixation biases that arise when individuals view social interactions that vary with regards to violence. Novel to this study is the use of scenes in which people are actively interacting, including explicitly violent social interactions. Previous studies that used violent stimuli have rarely included images depicting clearly malicious harm from a perpetrator to a victim^26^. Realistic stimuli are important if researchers wish to capture and study ecologically valid behavioral and emotional reactions to violence. The use of real images of violence that clearly display a perpetrator and victim in this study helped to mitigate this issue.

The aim of this study was to characterize gaze patterns associated with viewing violent and non-violent images of social interactions. In order to understand and gather information from a social interaction, the most relevant information may not be found on an individual’s body as a whole, but rather in particular aspects of the body that convey information about the interaction. We assumed that in a social interaction, faces, points of contact between people, and objects being used can be attended to as a means to infer social information such as actor intention. While there is a wealth of literature on the importance of faces for visual attention, the importance of contact points and objects being held have not been investigated as thoroughly. Still, some insightful work has been done on these areas, showing, for example, that objects can be important attractors of attention in natural scenes^27^. Moreover, specific kinematic features of hands are used to predict actor intentions^28^ and meaningful social interactions, such as a handshake, have been shown to attract and constrain attention in the same way that objects do^29^. Therefore, in addition to faces, we also measured gaze fixation to points of contact and objects people were holding. We assumed that in social interactions, and especially in violent ones, objects being held and points of contact would be good sources of information about the intentions and actions of the two people in the scene. To summarize, we analyzed gaze fixation on three points of interest in violent and non-violent social interactions: Faces, points of contact, and objects being held. Depending on the nature of the social interaction, we expected attention to be allocated differentially to these three areas.

## Results

### Descriptive statistics

We collected data from 6480 unique trials (72 images x 90 participants). After discarding trials in which total fixation time was less than 3000 ms, we ended up with 6236 unique trials for the full set of 72 images and 1129 unique trials for the 13 images that contained all three AOIs. See Table 1 for the average violence rating by interaction type.

**Table 1:** Descriptive statistics for dataset.

Interaction type	All Images			Images with all three AOIs		
	Violence rating *M* (*sd*)	Stimuli (*n*)	Trials (*n*)	Violence rating *M* (*sd*)	Stimuli (*n*)	Trials (*n*)
Friendly	1.10 (0.39)	24	2078	1.07 (0.34)	6	523
Ambiguous	1.87 (1.19)	24	2076	2.34 (1.35)	2	172
Violent	4.13 (1.53)	24	2082	4.15 (1.44)	5	434
Total	2.37 (1.72)	72	6236	2.45 (1.77)	13	1129

Mean violence rating, number of stimuli, and number of trials for each interaction type in both the full dataset and images with all three areas of interest.

### Simple LMM for dwell time on faces by interaction type

**Figure 1:**
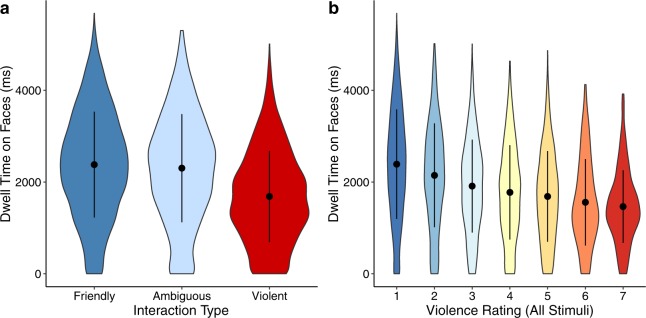
Attention to faces. (**a,b**) Participants spent less time looking at the faces of people engaged in a violent interaction, whether this was defined categorically by the researchers (**a**) or on a rating scale of perceived violence by the participant (**b**). Error bars represent ±1 *SD*.

Our main effect of interest in this study was to determine how visual attention to faces, contact points, and objects being held by the two main actors in an interaction varies depending on whether the social interaction being observed was friendly, ambiguous, or violent. To test this, we first conducted a trial-level linear-mixed model with interaction type (friendly, ambiguous, and violent) as the predictor, dwell time on faces as outcome, and participant ID and image ID as random effects. We found that participants spent less time looking at faces in violent interactions than in friendly (*b* = −695.34, 95% CI [−1022.66, −368.03], t = −4.220, *p* < 0.001) or ambiguous (*b* = −623.23, 95% CI [−950.56, −295.92], t = −3.782, *p* < 0.001) interactions (Fig. 1a).

### Complex LMM for dwell time on faces by interaction type

Because we used non-digitally altered images of real social interactions, the size and physical saliency of some of the AOIs differed. To account for this, we included physical saliency of faces and area of faces in pixels as fixed effects in our model. When controlling for physical saliency of the faces and the size of the AOIs, we still found that participants spent significantly less time looking at faces when interactions were violent compared to when they were friendly (*b* = −366.54, 95% CI [−622.12, −110.96], t = −2.849, *p* = 0.006) or ambiguous (*b* = −325.15, 95% CI [−578.34, −71.98], t = −2.551, *p* = 0.013). Still, other factors could be affecting dwell time. For instance, some images contained background faces, objects being held, or points of contact between individuals. To further test the strength of the relationship between dwell time on faces and violence, we included the area in pixels of other faces, objects being held, and contact points as fixed effects in the model. To summarize, (1) the predictor variable was interaction type; (2) the outcome was dwell time on faces; (3) fixed effects were saliency of face AOIs, area of face AOIs, area of background face AOIs, area of contact point AOIs and area of object AOIs; and (4) random effects were participant ID and image ID. Supporting our previous findings, this model showed that participants looked at faces significantly less when the interaction was violent than when it was friendly (*b* = −362.74, 95% CI [−600.12, −125.37], t = −3.036, *p* = 0.003) but not when it was ambiguous (*b* = −203.54, 95% CI [−449.54, 42.40], t = −1.644, *p* = 0.105), though the trend was in the same direction.

### Dwell time on faces by violence rating

It is possible that the broad category of interaction type as predefined by the researchers may obfuscate trends that occur as a function of perceived violence by the participant. In other words, an individual’s perception of how violent an interaction is might also be related to visual attention. Since participants rated each interaction for how violent it was on a 7-point scale, we should see a similar negative trend of dwell time on faces by violence rating. To test this, we used the same model as we did previously with interaction type. In other words, 1) the predictor variable was violence rating; 2) the outcome was dwell time on faces; 3) fixed effects were saliency of face AOIs, area of face AOIs, area of background face AOIs, area of contact point AOIs and area of object AOIs; and 4) random effects were participant ID and image ID. Supporting the results by interaction type, we found that participants spent less time looking at faces when they rated interactions as more violent (*b* = −45.00, 95% CI [−90.13, 0.41], t = −1.978, *p* = 0.048; Fig. 1b).

### Dwell time on faces in images with all 3 AOIs

**Figure 2:**
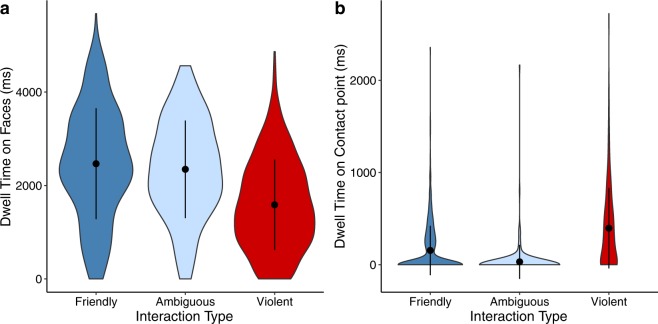
Attention to faces and points of contact in images with all three AOIs. (**a,b**) Even among images that contained all three areas of interest (contact points, objects being held, and faces), participants allocated less attention to faces (**a)** and more attention to contact points (**b**) in violent interactions. Error bars represent ±1 *SD*.

A subset of our images contained each area of interest (Table 1). While we control for the area of these AOIs in our models, with the area being 0 if it’s absent, it is possible that the presence of these AOIs alters attention in ways that our model cannot capture. Therefore, we also ran our full LMM for dwell time on faces by interaction type on the subset of data that contained all 3 AOIs. Because we only had two ambiguous images with all 3 AOIs, we did not think the results of statistical analysis on these images would be generalizable. Due to this and the fact that we were mostly interested in the differences between violent and non-violent interactions, we only compared friendly and violent interactions when using the subset of images with all 3 AOIs (however, we do include the data from the ambiguous images in Fig. 2). As in the full dataset, participants spent less time looking at faces in violent interactions than friendly interactions (*b* = −563.19, 95% CI [−879.00, −247.41], t = −3.775, *p* = 0.002; Fig. 2a). Likewise, violence rating negatively predicted dwell time on faces (*b* = −91.44, 95% CI [−140.16, −35.28], t = −3.837, *p* < 0.001).

### Dwell time on contact points

We predicted that contact points would be important sources of information in social interactions, especially in violent interactions. We measured attention to contact points in images with all 3 AOIs using an LMM where the predictor variable was interaction type, fixed effects were the saliency of contact points and area in pixels of each AOI, and random effects were participant ID and image ID. We found that participants looked significantly longer at contact points in violent interactions than in friendly interactions (*b* = 246.19, 95% CI [209.69, 318.69], t = 9.506, *p* < 0.001; Fig. 2b). Surprisingly, we did not find a relationship between violence rating and dwell time on contact points (*b* = 0.66, 95% CI [−21.03, 22.66], t = 0.067, *p* = 0.946).

### Dwell time on objects being held

We also thought that objects being held would be important sources of information in social interactions. We measured attention to objects being held in images with all 3 AOIs using an LMM where the predictor variable was interaction type, fixed effects were the saliency of objects being held and area in pixels of each AOI, and random effects were participant ID and image ID. While participants did spend a non-trivial amount of time looking at objects being held, this did not differ significantly between violent and friendly interactions (*b* = 423.18, 95% CI [−100.07, 946.44], t = 1.710, *p* = 0.111). Likewise, we did not find a relationship between violence rating and dwell time on contact points (*b* = 29.63, 95% CI [−4.30, 63.42], t = 1.736, *p* = 0.083), though the trend was clearer.

### First fixation analysis on faces

While we were mainly interested in total fixation time on our AOIs, it is also interesting to see where participants look first in a scene. In particular, we were interested to see if an initial saccade to faces would be less likely in violent interactions. We conducted an LMM with interaction type as the predictor and first fixation on faces as the outcome. The area and saliency of the face AOIs as well as the area of the other AOIs were included as fixed effects in the model with participant ID and image ID as random effects. Supporting our main findings, participants were less likely to fixate first on a face in violent interactions than in friendly interactions (*b* = −0.12, 95% CI [−0.22, −0.01], t = −2.202, *p* = 0.031). However, this relationship was not significant for violence rating (*b* = −0.00, 95% CI [−0.02, 0.01], t = −0.952, *p* = 0.341).

### First fixation analysis on contact points and objects being held

We were also interested in whether or not first fixation to contact points or objects being held was different in violent interactions. First, we conducted an LMM with interaction type as the predictor and first fixation on contact points as the outcome. The area and saliency of the contact point AOIs as well as the area of the other AOIs were included as fixed effects in the model with participant ID and image ID as random effects. As with the main analysis, we ran the LMM for contact points and objects on the subset of images that contained both of those AOIs. We found that participants did not fixate on contact points first more often in violent interactions than in friendly interactions (*b* = 0.01, 95% CI [−0.01, 0.03], t = 1.118, *p* = 0.285). Likewise, we did not find a relationship between the first fixation on contact point and violence rating (*b* = 0.00, 95% CI [−0.00, 0.01], t = 0.852, *p* = 0.401). We conducted the same analysis on objects being held and found that participants did not fixate first on objects more often in violent interactions than friendly (*b* = 0.01, 95% CI [−0.01, 0.03], t = 1.125, *p* = 0.281). We also found no relationship between violence rating and fixating first on an object being held (*b* = 0.02, 95% CI [−0.00, 0.01], t = 0.758, *p* = 0.454). These results suggest that attention biases towards contact points are not present from the onset of scene-viewing, but instead develop over the course of scene-viewing.

## Discussion

The purpose of this study was to characterize overt attention deployment when individuals viewed images depicting violent and non-violent social interactions. To this end, we analyzed attention to three regions of an interaction that we assumed to be important areas from which visual information about the intentions and actions of the interactors could be gathered. These three regions were the faces of the individuals who were interacting in the scene, contact points between them, and objects held in their hands.

The relative amount of attention allocated to these regions varied with respect to how violent the interaction was. While participants allocated significantly less attention to faces in violent interactions, they allocated significantly more attention to contact points. Moreover, the reduced attention to faces appears to be present from the onset of scene viewing, as evidenced by the first fixation analysis. Interestingly, we did not find a relationship between attention to objects being held and violence. This finding is at odds with the literature on the weapon focus effect, which suggests that weapons draw attention away from faces^30^. However, this line of research has largely focused on decision-making and memory rather than visual attention or information gathering per se. One possibility for why we did not see a category difference in attention to objects being held in our study is that our stimuli were socially and visually complex. Given this possibility, future research on the weapons focus effect should be sure to include socially complex stimuli.

Our use of active social scenes led to another interesting finding that diverges from previous research. Participants in our study spent much less time looking at faces than would be expected based on previous studies. We found that participants only spent about 34% of viewing time per image looking at the faces in violent interactions (Supplementary Table S1). What’s more, even in clearly non-violent (friendly) interactions, participants only spent about 48% of viewing time per image looking at faces — far less than previous studies that used real-world social scenes. For example, previous work on social attention in real-world scenes suggested that participants spent between 80 and 90% of viewing time on the eye and head regions combined^14^. While it is possible that some of the differences may be attributed to the amount of time the participants looked at each image in the two studies (6 s in the current study, 15 s in the previous study), the results of the current study point to the need to include a greater variety of active stimuli in social scene research.

On a larger scale, our findings support the notion that people don’t allocate attention to social interactions at random, nor do they just fixate on eyes or faces. Instead, we provide evidence for the claim that the implicit goal when viewing a social interaction is to gather relevant information about the individuals and the nature of their interaction and their intentions. This information is particularly important when witnessing violence, which is an opportunity to gather socially critical information about aggressors and victims, potential coalitional partners, and changes in status, power, and hierarchy.

One limitation of this study was that our images mostly involved two main individuals interacting. While dyadic interactions are common in real life, it is not uncommon for more than two people to interact. It is unclear how visual patterns would change when there are three or more people interacting. However, it is clear that participants attended considerably more to the main faces of the scene than background faces, which garnered a negligible amount of dwell time (Supplementary Table S1). While the main point of this study was to see the difference between violent and non-violent images, our stimuli also included ambiguous images. However, we lacked enough ambiguous images that contained objects and contact points, which limited our ability to draw strong inferences about this category of interaction. It is possible that one feature common to ambiguous interactions is the lack of objects and contact points, which might be key features that are used to make meaning out of an interaction. Another feature that we assumed would be important was symbolic information, which could provide additional context or information about interaction or the individuals (e.g., name badges, signs, words on clothing). Indeed, previous research suggests that text in a scene can attract gaze^31^. In our study, 12 of the friendly images, 9 of the ambiguous images, and 6 of the violent images contained text. However, the complex nature of our stimuli made symbolic information difficult to operationalize in our study, so we did not analyze it. More specifically, it was unclear whether we should include and lump together symbols such as logos, badges, and all text, regardless of size, as “symbolic information”. Thus, we decided it would be best to leave this topic for a future study that is designed to test this interesting question.

While the images we gathered benefitted from being real images of social interactions, the experimental control over the stimuli was limited. However, we attempted to statistically control for this by including physical saliency and AOI size as fixed effects and a random intercept for each image in our mixed models. Still, previous studies have reported gaze differences between real life situations and closely matched laboratory conditions^32^. It remains unclear whether or not the gaze patterns reported here would replicate in real life situations, though this would be difficult to test considering the topic. While sports such as boxing or MMA might seem like a plausible way to test this question using mobile eye tracking, there could be a confound with participants being distracted by technique or particular moves. One way around this could be to record participants’ gaze using mobile eye tracking while they view violence that occurs outside of a sport context, such as violence in theatrical plays.

Although our participant sample was not completely homogenous, many of the participants were undergraduate students. While some visual biases involved in information gathering from violent social interactions are likely stable across individuals, it is possible that a more diverse sample would produce different results. Likewise, there likely exist some individual differences that affect viewing patterns in violence. For example, previous research has suggested that traits such as anger can affect the interpretation of potentially hostile acts^33^. The theoretical and empirical implications of this research could have important consequences for the criminal justice system, where video evidence of violent interactions is sometimes used by juries to make decisions about guilt or innocence. If particular features of an image can strongly influence viewing behavior and interpretation of the action as more or less violent, then it is critical to understand how the content of images or videos shown in courtrooms affect viewing behavior and, ultimately, interpretations of scenes. It is important to understand not only general trends in viewing behavior, but also the effect of emotional states and individual differences. While a few studies have looked at the role of gaze allocation and individual differences in attributions of intent^34,35^, there is still much to learn about the effect of content on gaze allocation to particular features and interpretation of violent scenes.

Although it has been demonstrated that humans possess visual biases that aid in gathering relevant information from stimuli in the environment, research in this area had not previously been extended to an unfortunately common phenomenon in human social life – violence. Given the prevalence of violence throughout human history, we know remarkably little about how people gather information from scenes of violence. Since witnessing interpersonal violence provides a unique opportunity to gather socially and ecologically critical information, visual attention to certain features might be biased when viewing violent interactions. This study is an important first step in defining key attentional features of viewing violent social interactions. We demonstrated that attention to faces is greatly diminished when an interaction is perceived as violent, and that at least some of the attention that is diverted away from faces when viewing violent interactions is directed toward points of contact. Participants attended to contact points significantly more when viewing violent interactions, suggesting that they may contain information that is important for discerning the intentions and actions of individuals engaged in violence.

## Method

### Identifying information

Informed consent was obtained for the publication of identifying information/images in an online open-access publication.

### Participants

The study was approved by the Social Sciences Institutional Review Board at the University of Chicago and all methods were carried out in accordance with the relevant guidelines and regulations. A sample size between 75 and 93 was determined to detect a small-medium effect size (*d* = 0.3) between three groups with 90–95% power using a repeated measures ANOVA. Because we were recruiting during the school year, we decided to stop at the end of an academic quarter where the number of participants fell within this range. Though we initially planned on doing ANOVA for analyses, we eventually decided to use linear-mixed modeling for a more rigorous statistical analysis. A post-hoc power analysis suggests that our power to detect the effect that we observed of interaction type on dwell time was 99%. Informed consent was obtained from all participants (*n* = 90), who were mainly college students recruited through online ads. Fifty-six females and 30 males completed the demographics (age range: 18–28, median 20). Self-identified races were White (49%), Asian (28%), Black (9%), Hispanic (6%), and multiple races (8%). All participants spoke fluent English and had normal or corrected-to-normal vision.

### Stimuli

Participants viewed 72 full-color images sized at 1600 × 900 pixels. All images were obtained from online news and media sources and consisted of photos showing interactions between two adult males. Previous research suggests that males are more likely to be both the perpetrators and the victims of violence than females^36^. Images were selected so that 1/3 of them (i.e., 24 images) represented violent interactions, 1/3 represented friendly interactions, and 1/3 represented ambiguous (neither clearly violent nor clearly friendly) interactions. To check that the violent images were rated as more violent than the ambiguous and friendly images, all images were rated for how violent they were on a 9-point scale in an MTurk pilot prior to the study. This pilot was simply a check to make sure images were categorized well. Violent images were rated an average of 6.8, ambiguous images were rated an average of 3.6, and friendly images were rated an average of 1.8, suggesting that the images were categorized appropriately.

### Experimental apparatus and procedures

Participants sat 95 cm away from a 24″ LCD monitor with a 1920 × 1080 pixels resolution and viewed the images that were displayed at the center in their native resolution; 60 pixels corresponded to one degree in visual angle. Stimuli were presented using MATLAB with the Psychophysics Toolbox extension^37–39^. Eye movements were recorded from both eyes using an SR Research (Ottawa, Ontario, Canada) Eyelink 1000 eye tracker in head free-to-move remote mode with a sampling rate of 500 Hz.

Once seated, participants signed the consent form, performed the eye tracker calibration (using a nine-point calibration routine) and validation, and then performed a practice block consisting of six images not used in the study. In the main blocks, the 72 images were split into four blocks of 18, and the split and order was randomized for each participant. Participants were told they would be viewing a series of images that contained social interactions. They were asked to view each image naturally, as if viewing them online. In each trial, participants clicked a central dot (diameter: 0.3° in visual angle) to begin. This forced participants to fixate at the screen center for fine visuomotor coordination and thus allowed us to inspect the quality of eye tracking on a trial-by-trial basis (Supplementary Fig. S1). The mean and covariance matrix of the participants’ averaged-across-trial first fixations (Supplementary Fig. S2) were [horizontal: −0.31°, vertical: 0.33°] and [0.76, −0.27; −0.27, 0.91], respectively. Although the quality of eye tracking differed by participant, no participant was excluded. After clicking the dot, participants viewed an image for 6 seconds while their eye movements were recorded. After the image disappeared, participants rated how violent the interaction in the image was on a 7-point scale (1 = *not violent*, 7 = *extremely violent*). After rating, participants were asked to verbally describe the social interaction while inspecting the same image again for 12 seconds. The verbal report data are not presented here, but are being analyzed for another manuscript. After each block of images, participants were asked to briefly close their eyes to help prevent fatigue.

### Areas of interest (AOIs)

We selected three AOIs that we assumed were important for understanding social interactions. Based on previous literature, we reasoned that the *faces* of the two individuals who are interacting would be the most attended area. However, we also anticipated that two other areas — *points of contact* between the two individuals and *objects* being held — would convey important information for understanding the individuals’ intentions and actions. The AOIs were defined by drawing an oval around the face, contact point, or object being held using a custom GUI tool in MATLAB (see Supplementary Fig. S3 for an example of this). Out of 72 images, 40 contained a point of contact AOI, 22 contained an object AOI, and 13 contained both AOIs.

### Eye movement analysis

Fixation data were preprocessed using Eyelink Data Viewer v2.2.1 (SR Research, Ottawa, Canada). The first fixation of each trial (Supplementary Fig. S1) was discarded, as those central fixations were carried over from the pre-stimulus period. Trials where total fixation time was less than 3000 ms (half of stimulus-presentation time) were discarded, as this suggests measurement error due to technical problems or participants’ inattention. Fixations were drift-corrected by subtracting the distance of the first fixation from the location of the central fixation cross to take into account the measurement errors of video-based eye trackers^40,41^. The AOI-level dwell time data were summarized using custom MATLAB scripts. Dwell time was calculated for the AOIs by summing the duration of fixations that fell inside the oval that defined the AOI. If there was more than one AOI of a kind (e.g., two faces), each trial’s dwell time was the sum of dwell times of each AOI in that category.

### Statistical analysis

Trial-level linear mixed effects models (LMM) were run using the lme4 package in R^42^. LMM allows for isolation of effects of interest, such as violence rating in this study, while simultaneously controlling for differences between stimuli and participants^43^. As such, the by-participant and by-image random intercepts were included as random effects. We also included as fixed effects the size in pixels of all AOIs and physical saliency of the AOI of interest. Physical saliency was determined using the Graph-Based Visual Saliency (GBVS) algorithm^44^. Confidence intervals for model parameters were obtained using the *confint* function in R. Model statistics, including marginal and conditional R^2^, are summarized in Supplementary Table S2.

## Supplementary information

Supplementary Information: Violence reduces attention to faces and draws attention to points of contact

## Data Availability

All stimuli, data, and analysis code are available at https://osf.io/sfyj2/.
