# Supplementary material for: Violence reduces attention to faces and draws attention to points of contact
Source: Sci Rep. 2019 Nov 28;9:17779. doi: 10.1038/s41598-019-54327-3 (PMC6883035; doi:10.1038/s41598-019-54327-3)
Supplement: Supplementary file 1 — Supplementary Information: Violence reduces attention to faces and draws attention to points of contact [file 41598_2019_54327_MOESM1_ESM.pdf]

Supplementary Information: Violence reduces attention to faces and draws attention to points of contact

Coltan Scrivner*[1,2], Kyoung whan Choe[3,4], Joseph Henry[2,3], Muxuan Lyu[3], Dario Maestripieri[1,2], and Marc G. Berman[3,5]

[1]Department of Comparative Human Development, The University of Chicago, Chicago, IL, USA

[2]Institute for Mind and Biology, The University of Chicago, Chicago, IL, USA

[3]Department of Psychology, The University of Chicago, Chicago, IL, USA

[4]Mansueto Institute for Urban Innovation, The University of Chicago, Chicago, IL, USA

[5]Grossman Institute for Neuroscience, Quantitative Biology, and Human Behavior

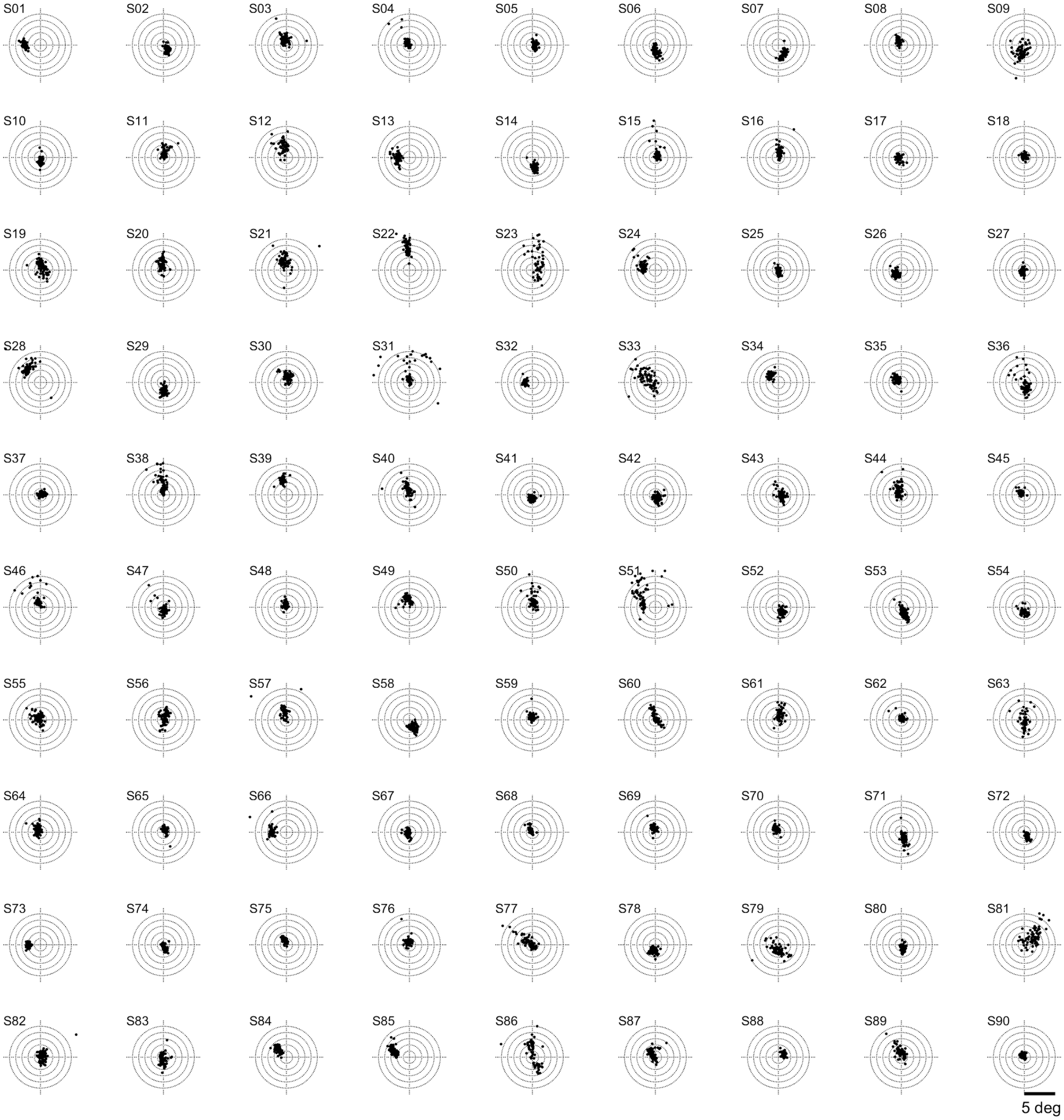

Fig. S1. The first fixations of all participants. Each dot represents a trial. The dotted horizontal and vertical lines represent the horizontal and vertical centers of the screen, respectively, and the dotted circles indicate isodistance zones from the screen center in 1° visual angle increments. No participants were excluded in our analyses.

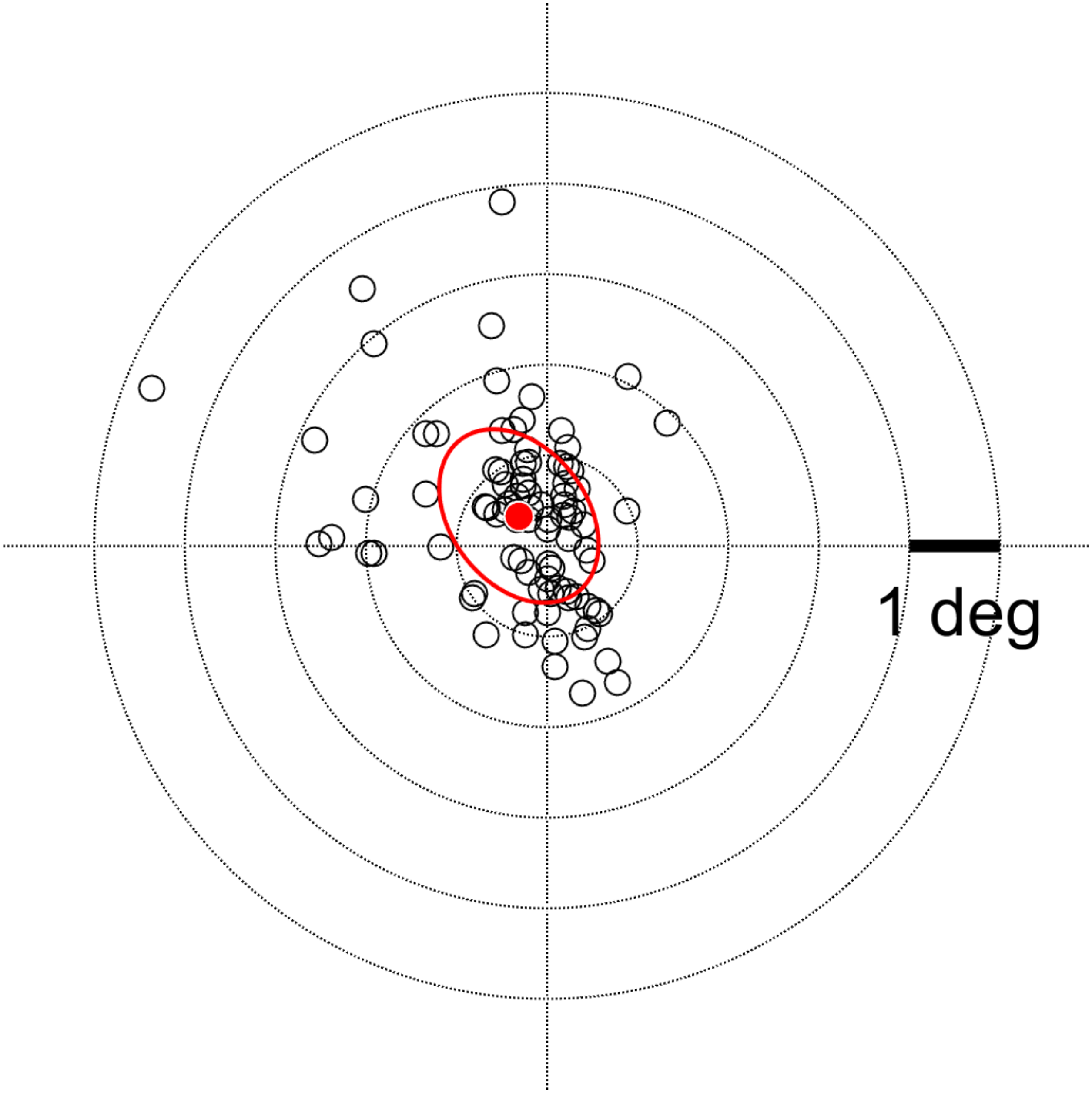

Fig. S2. The averaged-across-trial first fixations of all participants. Each open circle represents a participant. The red filled circle and the red ellipse represent the mean (horizontal = -0.31°, vertical = 0.33°) and standard deviation (covariance matrix = [0.76, -0.27; -0.27, 0.91]), respectively, of the participants' averaged-across-trial first fixations.

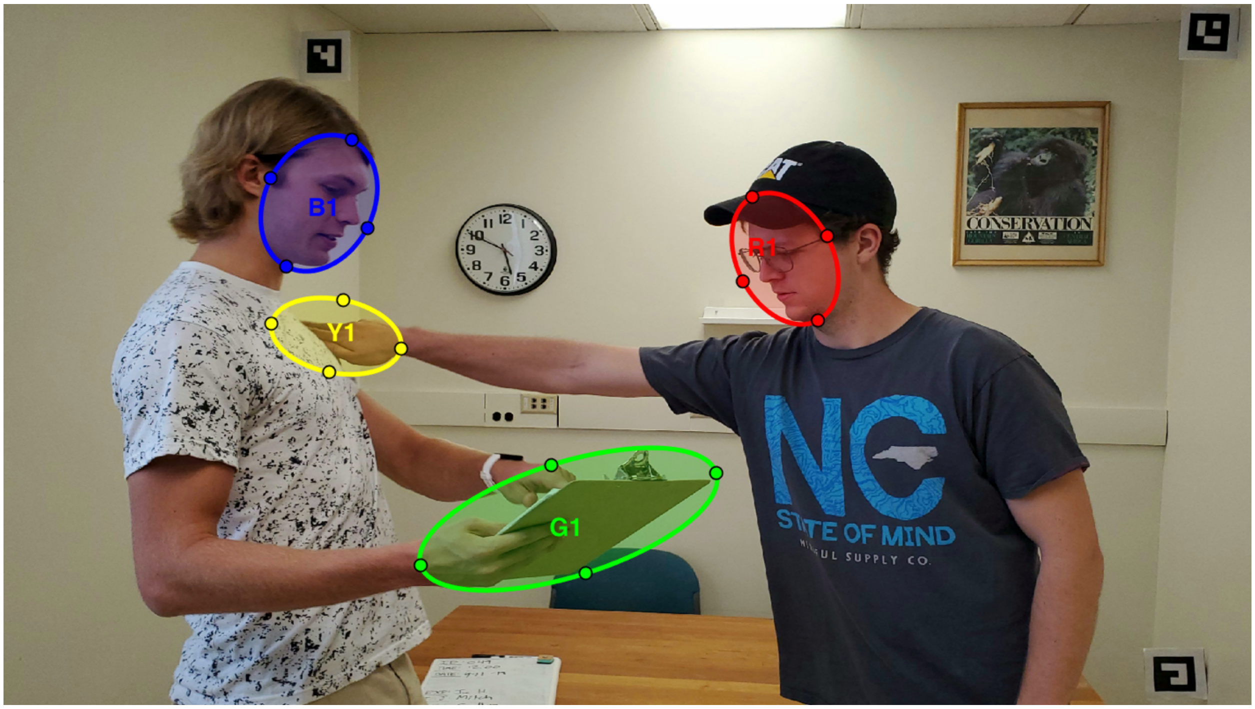

Fig. S3. Example image with AOIs drawn that was not used in the study. The blue B1 and red R1 ovals are the face AOIs, the yellow Y1 is the contact point AOI, and the green G1 is the object being held AOI.

Model	*b*	Lower CI	Upper CI	t	*p*	Marginal R2	Conditional R2
Simple LMM: Dwell on faces by Interaction Type	-695.34	-1022.66	-368.03	-4.220	<.001	.07	.44
Semi-complex LMM: Dwell on faces by Interaction Type	-366.54	-622.12	-110.96	-2.849	.006	.19	.44
Complex LMM: Dwell on faces by Interaction Type	-362.74	-600.12	-125.37	-3.036	.003	.20	.44
Complex LMM: Dwell on faces by violence rating	-45.00	-90.13	0.41	-1.978	.048	.19	.44
3 AOIs: Dwell on faces by interaction type	-563.19	-879.00	-247.41	-3.775	.002	.33	.44
3 AOIs: Dwell on faces by violence rating	-91.44	-140.16	-35.28	-3.837	<.001	.33	.44
3 AOIs: Dwell on contact by interaction type	246.19	209.69	318.69	9.506	<.001	.23	.23
3 AOIs: Dwell on contact by violence rating	0.66	-21.03	22.66	0.067	.946	.12	.23
3 AOIs: Dwell on object by interaction type	423.18	-100.07	946.44	1.710	.111	.28	.48
3 AOIs: Dwell on object by violence rating	29.63	-4.30	63.42	1.736	.083	.24	.46
First fixation on face by interaction type	-0.12	-0.22	-0.01	-2.202	.031	.11	.28
First fixation on face by violence rating	0.00	-0.02	0.01	-0.952	.341	.10	.28
First fixation on contact by interaction type	0.01	-0.01	0.03	1.118	.285	.03	.03
First fixation on contact by violence rating	0.00	0.00	0.01	0.852	.401	.03	.03
First fixation on object by interaction type	0.01	-0.01	0.03	1.125	.281	.03	.03
First fixation on object by violence rating	0.02	0.00	0.01	0.758	.454	.03	.03

Table S1. Stats for linear mixed models. For models with interaction type as the IV, this table shows the effect of violent images as compared to friendly images. Simple LMM means only the DV of interest (dwell time on faces) was included as a fixed effect. In the semi-complex model, area in pixels and physical saliency of faces were controlled for as fixed effects. In the complex LMM, area of the other AOIs were added into the model as fixed effects. Participant ID and Image were included as random effects in all models. Beta for first fixation is mostly uninterpretable because it is simply 0 or 1 for absence or presence of first fixation on the AOI.

		Friendly Images	Violent Images	All images
Faces (All images)	Mean (ms)	2381	1685	2122
	Percent of Total	48%	34%	43%
	First Fixation	54%	32%	47%
Faces (including background)	Mean (ms)	2520	1795	2251
	Percent of Total	51%	34%	45%
	First Fixation	56%	32%	47%
Faces (When 3 AOIs)	Mean (ms)	2466	1588	2110
	Percent of Total	50%	32%	43%
	First Fixation	57%	24%	48%
Contact Points	Mean (ms)	155	396	229
	Percent	3%	8%	5%
	First Fixation	0%	3%	1%
Objects	Mean (ms)	601	646	570
	Percent of Total	12%	13%	11%
	First Fixation	2%	9%	4%

Table S2. Mean and percentage of total viewing time. The mean dwell time (ms), percent of viewing time, and percent of total first fixations attributed to each AOI by image type. Numbers for contact points and objects are only in images with all 3 AOIs. “Faces (including background)” is the dwell time and percentages when including fixations on background faces. As seen in the table, background faces make little to no difference in fixation time or number of first fixations.
